# Six new dactylogyrid species (Platyhelminthes, Monogenea) from the gills of cichlids (Teleostei, Cichliformes) from the Lower Congo Basin

**DOI:** 10.1051/parasite/2018059

**Published:** 2018-12-07

**Authors:** Michiel W.P. Jorissen, Antoine Pariselle, Tine Huyse, Emmanuel J. Vreven, Jos Snoeks, Eva Decru, Thomas Kusters, Soleil Wamuini Lunkayilakio, Fidel Muterezi Bukinga, Tom Artois, Maarten P.M. Vanhove

**Affiliations:** 1 Research Group Zoology: Biodiversity, & Toxicology, Centre for Environmental Sciences, Hasselt University Agoralaan gebouw D 3590 Diepenbeek Belgium; 2 Royal Museum for Central Africa Leuvensesteenweg 13 3080 Tervuren Belgium; 3 ISEM, Univ de Montpellier, CNRS, IRD Montpellier France; 4 Laboratory of Biodiversity and Evolutionary Genomics, University of Leuven Charles Deberiotstraat 42 3000 Leuven Belgium; 5 Institut Supérieur Pédagogique Mbanza-Ngungu B.P. 127 Democratic Republic of the Congo; 6 Centre de Recherche en Hydrobiologie Uvira B.P. 73 Democratic Republic of the Congo; 7 Department of Botany and Zoology, Faculty of Science, Masaryk University Kamenice 735/5 625 00 Brno Czech Republic; 8 Zoology Unit, Finnish Museum of Natural History, University of Helsinki Pohjoinen Rautatiekatu 13 00100 Helsinki Finland

**Keywords:** Parasitology, biodiversity, biogeography, *Cichlidogyrus*, Cichlidae

## Abstract

The Lower Congo Basin is characterized by a mangrove-lined estuary at its mouth and, further upstream, by many hydrogeographical barriers such as rapids and narrow gorges. Five localities in the mangroves and four from (upstream) left bank tributaries or pools were sampled. On the gills of *Coptodon tholloni*, *Coptodon rendalli*, *Hemichromis elongatus*, *Hemichromis stellifer* and *Tylochromis praecox*, 17 species of parasites (Dactylogyridae & Gyrodactylidae, Monogenea) were found, eight of which are new to science. Six of these are herein described: *Cichlidogyrus bixlerzavalai* n. sp. and *Cichlidogyrus omari* n. sp. from *T. praecox*, *Cichlidogyrus calycinus* n. sp. and *Cichlidogyrus polyenso* n. sp. from *H. elongatus*, *Cichlidogyrus kmentovae* n. sp. from *H. stellifer* and *Onchobdella ximenae* n. sp. from both species of *Hemichromis*. On *Cichlidogyrus reversati* a ridge on the accessory piece was discovered that connects to the basal bulb of the penis. We report a putative spillback effect of the native parasites *Cichlidogyrus berradae*, *Cichlidogyrus cubitus* and *Cichlidogyrus flexicolpos* from *C. tholloni* to the introduced *C. rendalli*. From our results, we note that the parasite fauna of Lower Congo has a higher affinity with the fauna of West African and nearby freshwater ecoregions than it has with fauna of other regions of the Congo Basin and Central Africa.

## Abstract (fr)

Le cours du Bas Congo est caractérisé par un estuaire bordé de mangroves et, plus en amont, par de nombreuses barrières hydro-géographiques formées de rapides ou de gorges étroites. Nous avons échantillonné dans cinq localités au niveau des mangroves et dans quatre autres en amont sur la rive gauche du fleuve, dans des affluents ou des étangs. Nous avons trouvé, sur les branchies de *Coptodon tholloni*, *Coptodon rendalli*, *Hemichromis elongatus*, *Hemichromis stellifer* et *Tylochromis praecox*, 17 espèces de parasites (Dactylogyridae et Gyrodactylidae, Monogenea), dont huit sont nouvelles pour la science. Six d’entre elles sont décrites ici : *Cichlidogyrus bixlerzavalai* n. sp. et *Cichlidogyrus omari* n. sp. sur *T. praecox*, *Cichlidogyrus calycinus* n. sp. et *Cichlidogyrus polyenso* n. sp. sur *H. elongatus*, *Cichlidogyrus kmentovae* n. sp. sur *H. stellifer* et *Onchobdella ximenae* n. sp. sur les deux espèces d’*Hemichromis*. Nous décrivons, chez *Cichlidogyrus reversati*, une arête sur la pièce accessoire qui la connecte avec l’ampoule basale basal du pénis. Nous notons un probable transfert latéral des espèces autochtones *Cichlidogyrus berradae*, *Cichlidogyrus cubitus* et *Cichlidogyrus flexicolpos* de *C. tholloni* vers l’espèce introduite *C. rendalli*. Nos résultats montrent que la faune parasitaire du Bas Congo présente plus d’affinités avec les faunes d’Afrique de l’Ouest ou des écorégions d’eau douce voisines, qu’avec celles des autres parties du bassin du Congo ou de l’Afrique Centrale.

## Introduction

**Figure 1.**
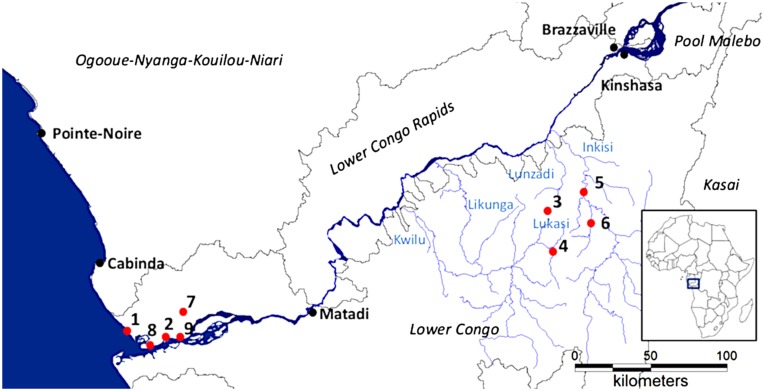
Overview of sampling localities (red dots) with the ecoregions in italics. Localities 3 and 5 are located within the cities Mbanza-Ngungu and Kisantu, respectively. The inset shows the Lower Congo region on the African continent. Sampling localities: 1, Tondé Estuary near Muanda (05°57′35.9″S 12°21′58.4″E); 2, Muila Kaku, mangroves near Lower Congo River (05°59′33″S 12°35′03.2″E); 3, Ndimba Leta ponds, Mbanza-Ngungu, in between the Lunzadi and Lukasi rivers of which the latter flows into the Kwilu and Likunga (05°15′10″S 14°51′24″E); 4, Pond near Kila Kindinga and Lukasi river (5°29′7″S 14°53′4″E); 5, Inkisi River, off the Jardin botanique de Kisantu (05°08′02″S 15°03′52″E); 6, Mvuazi River, Inkisi (5°19′S 15°7′E); 7, Mbola River near Tshianya village (05°52′09.8″S 12°39′52.6″E); 8, Congo River near Nganda Flash station (06°02′01.8″S 12°31′48.2″E), 9, Muila Nzenze, Kibamba village, mangroves near Congo River (06°00′06.8″ S 12°40′27″ E). Rivers in blue.

The Lower Congo River (LCR) is 350 km long and extends from the Kintambo Rapids at the outlet of Pool Malebo to the Atlantic Ocean [2]. With its tributaries, the LCR forms the Lower Congo Basin, which contains the Lower Congo and the Lower Congo Rapids ecoregions. The Lower Congo ecoregion covers the LCR from its mouth to Matadi and the left-bank tributaries, the largest of which is the Inkisi River. At the mouth, a mangrove-lined estuary is found with euryhaline fishes and freshwater representatives of marine families. The Lower Congo Rapids ecoregion spans the LCR from Matadi to the Kintambo Rapids and the right-bank tributaries (see Fig. 1) [1]. The Lower Congo Rapids ecoregion is characterized by large rapids and canyons that are up to 200 m deep [24, 41]. This makes it the most hydrologically and spatially complex of the two ecoregions. Because of this complexity, there is a high habitat heterogeneity through numerous dispersal barriers. This and the downstream location in the Congo basin lead to the LCR having a hyperdiverse ichthyofauna with high levels of species endemism [15, 16]. Of the over 320 fish species, 84 are documented endemics for the LCR [15]. The families Mochokidae, Cyprinidae and Cichlidae are the most species-rich in the area [1]. The focus of this study is the taxonomic exploration of monogenean gill parasites (Platyhelminthes) of Cichlidae (Cichliformes: Teleostei) in the Lower Congo Basin. More specifically, representatives of *Cichlidogyrus* Paperna, 1960 (Dactylogyridae), *Onchobdella* Paperna, 1968 (Dactylogyridae) and *Gyrodactylus* Von Nordmann, 1832 (Gyrodactylidae) are likely to be observed, because they are known to infect African cichlids [34, 48]. Of these, *Cichlidogyrus*, with over 100 species described, is the most species-rich genus occurring on cichlids throughout Africa and the Middle East [14, 34, 39, 40]. Species of *Onchobdella* are known from species of *Hemichromis* Peters, 1857, from *Pelmatochromis buettikofferi* (Steindachner, 1894) and *Chromidotilapia guntheri* (Sauvage, 1882) [31]. *Onchobdella* comprises eight species [4, 26, 30]. They occur mostly in West Africa, but have also been found in Cameroon [34]. *Gyrodactylus* includes 17 species known from African cichlids. This genus, however, is much larger, as over 450 species have been described and representatives are known to infect most fish orders [12, 48]. The members of *Onchobdella*, *Cichlidogyrus* and *Gyrodactylus* are most easily distinguished through the morphology of the haptor, the caudal attachment organ, which includes sclerotized hooks and transverse bars. The haptor of species of *Cichlidogyrus* consists of seven pairs of hooks (I–VII), one pair of dorsal and one pair of ventral anchors associated with dorsal and ventral transverse bars. Hooks of pair II are always short and associated with the ventral anchors. Pairs I and III–VII can be short or elongated [32, 34, 46, 47]. The haptor of species of *Onchobdella* contains five to six pairs of short hooks, a pair of large dorsal anchors that are arranged distal-laterally, one pair of small ventral anchors associated with two ventral bars and one large horseshoe-shaped or straight dorsal bar [26, 34]. The hard parts in the haptor of species of *Gyrodactylus* comprise 16 marginal hooks and a pair of anchors that are connected by a superficial and deep ventral bar [38]. Species within these genera can be distinguished based on morphological differences between sclerotized structures, which are the male copulatory organ (MCO), the vagina (when sclerotized), and the haptoral sclerites. These parasite genera have not been thoroughly studied in the Lower Congo Basin, but parasites from the neighbouring Ogooué-Nyanga-Kouilou-Niari freshwater ecoregion (see Fig. 1) [1] were explored [30, 33]. These include *Cichlidogyrus reversati* Pariselle & Euzet, 2003 and *C. lemoallei* Pariselle & Euzet, 2003 from the mouth of the Lower Kouilou River (Republic of Congo, ROC), *C. berradae* Pariselle & Euzet, 2003 from Lake Cayo and Loufoualéba, and *C. legendrei* Pariselle & Euzet, 2003 and *Scutogyrus chikhii* Pariselle & Euzet, 1995 from Lake Cayo. These parasites were collected from *Pelmatolapia cabrae* (Boulenger, 1899), except for *Scutogyrus chikhii*, which was described from the introduced *Oreochromis mossambicus* (Peters, 1852). In addition, *Cichlidogyrus microscutus* Pariselle & Euzet, 1996 was found on *Coptodon guineensis* (Günther, 1862) from Lake Loufoualéba (see [32]). From the Congo River itself, *Cichlidogyrus flexicolpos* Pariselle & Euzet, 1995 was described from *Coptodon guineensis* [29]. This study serves as a first exploration of the gill monogeneans of *Coptodon tholloni* (Poll & Thys van den Audenaerde, 1960), *Hemichromis elongatus* (Guichenot, 1861), *Hemichromis stellifer* Loiselle, 1979 and *Tylochromis praecox* Stiassny, 1989. Parasites from *C. rendalli* have already been studied from the Bangwuelu-Mweru ecoregion [14, 46] and from Lake Kariba [7].

## Materials and methods

**Figure 2.**
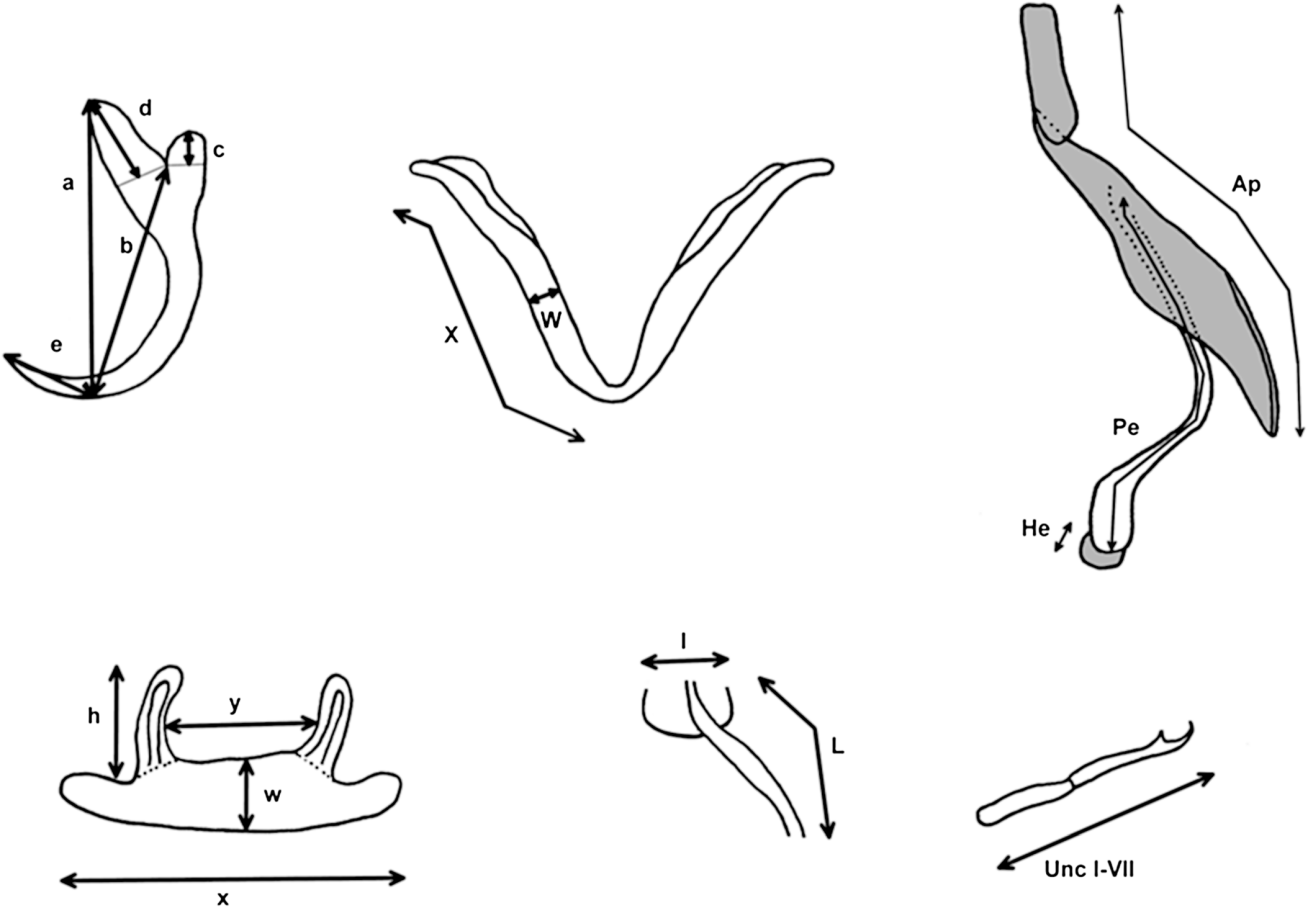
Schematic representation of the measurements taken of the hard parts of specimens of *Cichlidogyrus* and *Onchobdella*. Structures from top left to bottom right: anchor, ventral transverse bar, MCO, dorsal transverse bar, vagina and hook. Abbreviations: I–VII, hook length; a, total anchor length; Ap, length of accessory piece; b, blade length; c, shaft length; d, guard length, e, point length; h, auricle length; He, Heel length; l, vagina width; L, vagina length; Pe, penis length; W, maximum width of ventral transverse bar; w, maximum width of dorsal transverse bar; X, branch length of ventral transverse bar; x, total length of dorsal transverse bar; y, distance between auricles.

During a field expedition to the Lower Congo region in June 2015, fish were collected from nine localities (see Fig. 1 and caption for the coordinates). Fish were caught using gill nets and euthanized with an overdose of MS222. All specimens were diagnosed in the field and later verified in the lab, except for the specimen of *T. praecox*, which was verified in the lab with a picture of the specimen, because it came from an existing collection from the Institut Supérieur Pédagogique de Mbanza-Ngungu and was not transported to the RMCA, Royal Museum for Central Africa. From each fish, the right gill arches were removed, stored on ethanol and used for parasitological screening. The storage fluid was also exhaustively screened. Parasites were collected with an entomological needle under a WILD M5 stereomicroscope (Wild, Heerbrugg, Switzerland), mounted on a glass slide and fixed with Malmberg’s solution (in the field) or Hoyer’s medium (in the lab). Coverslips were sealed with Glyceel [3], D-pex or nail polish. Parasites were identified through a Leitz Dialux 22 microscope (Leitz, Wetzlar, Germany) with differential interference contrast and measured with Auto-montage software (Imaging & Microscopy, Weinheim, Germany). Images were taken with an optical camera on a Leica DM2500 microscope with Leica Application suite software V.X (Leica, Wetzlar, Germany). Illustrations were drawn using a drawing tube and finalized with GIMP V2.8 (www.gimp.org). The accessory piece and heel of the MCO are represented with a grey filling, to emphasize the plate-like structure and to distinguish it from the penis, which is a hollow duct. Measurements were carried out following Figure 2 and represented as average ± standard deviation, individuals measured (minimum value − maximum value). The standard deviation is only represented when *n* ≥ 30. Numbering of the hooks follows Pariselle & Euzet [33, 35]. Cichlids were deposited in the ichthyology collection of the RMCA in collections 2015–29 under accession numbers AB54005942, AB53952197 and AB54006866 and 2015–30 under RMCA_Vert_2015.030.P.0009–0022. Individual accession numbers are represented in addendum 1. Whole-mounted parasites were deposited in the collections of the RMCA (M.T.38280–38375), the Natural History Museum (London, United Kingdom, NHMUK 2018.1.31.1–6), the Iziko South African Museum (Cape Town, South Africa, SAMC-A090065–69) and the Finnish Museum of Natural History (Helsinki, Finland, MZH, KN10043–10057). The MZH specimens can be consulted online through https://laji.fi/en/view?uri=luomus:KN.10043 and onwards. More hyperlinks and all accession numbers of parasites are given in Addendum 2.

## Results

Seventeen species of monogeneans, eight of which are new to science, were found on five hosts (Table 1). For five described parasite species, this is their first record from the Lower Congo region, and for all of them, new host records were found. Six new species are described in the present study. In addition, morphological remarks are given for *C. reversati*, because the specimens in this study varied slightly from the original description. Additionally, more details on the connection of the accessory piece with the basal bulb of the penis in *C. reversati* were observed and mentioned under remarks.

**Table 1.** Occurrence of parasite species on their respective hosts, marked by an “x” and abundance of host and parasite, respectively.

	*C. tholloni*	*C. rendalli*	*H. elongatus*	*H. stellifer*	*T. praecox*	N (171)
N (14)	5	3	4	1	1	
C. berradae*		H				8
*C. bixlerzavalai* n. sp.*					H	3
*C. calycinus* n. sp.*			H			16
*C.* sp.1*	x					3
*C.* sp.2*	x					9
C. cubitus*	H	H				36
*C. falcifer*			H			11
*C. flexicolpos*		H				5
*C. polyenso* n. sp.*			H			7
*C. kmentovae* n. sp.*				H		5
*C. longicirrus*			H			1
*C. omari* n. sp.*					H	1
*C. reversati*	H					8
C. tilapiae*	H					4
*C.* sp.		x	x			4
G. chitandiri*	H					21
O. aframae*			H			3
*O. ximenae* n. sp.*			H	H		25
*O.* sp			x			1

* Represents the first record of this species in Lower Congo. An “H” represents a new host record. “N” represents the number of hosts (2nd row) or parasites (last column).

**Table 2.** Number of parasite species per locality and infection intensity. Localities correspond to those in Figure 1. See Addenda 1 and 2 for the individual voucher and accession numbers.

Host species	Parasite species	Locality	Infection intensity/no. infected hosts
*Coptodon tholloni*	*C.* sp.1	9	3/1
	*C.* sp.2	9	9/1
	*C. cubitus*	4	1/1
		5	1/1
		8	1/1
		9	5/1
	*C. reversati*	8	8/1
	*C. tilapiae*	3	4/1
	*G. chitandiri*	5	21/1
*Coptodon rendalli*	*C. berradae*	1	1–4/3
	*C. cubitus*	1	7–14/3
	*C. flexicolpos*	1	5/1
*Hemichromis elongatus*	*C. calycinus* n. sp.	4	3–6/3
		6	2/1
	*C. falcifer*	4	1–10/2
		6	1/1
	*C. polyenso* n. sp.	4	2–3/3
	*C. longicirrus*	4	1/1
	*O. aframae*	4	4/1
	*O. ximenae* n. sp.	4	1–8/1
		6	3/1
*Hemichromis stellifer*	*C. kmentovae* n. sp.	7	5/1
	*O. ximenae* n. sp.	7	1/1
*Tylochromis praecox*	*C. bixlerzavalai* n. sp.	2	4/1
	*C. omari* n. sp.	2	1/1

### *Cichlidogyrus bixlerzavalai* Jorissen, Pariselle & Vanhove n. sp.

urn:lsid:zoobank.org:act:3E7C4017-8E0E-4DE3-A3BD-6CD67D806601

Type host: *Tylochromis praecox* Stiassny, 1989.

Infection site: Gills.

Type locality: Muila Kaku, mangroves near Lower Congo River 05°59′33″S 12°35′03.2″E.

Material: Four whole-mounted specimens in Malmberg’s solution.

Holotype: M.T.38339.

Paratypes: M.T.38340–41, KN10053 https://laji.fi/en/view?uri=luomus:KN.10053.

Etymology: The species epithet is a homage to singer Cedric Bixler-Zavala of bands At the drive-in, The Mars Volta and Antemasque and is a noun (name) in the genitive case.

Authorship: Note that the authors of the new taxon are different from the authors of this paper; Article 50.1 and Recommendation 50A of International Code of Zoological Nomenclature [13].

#### Description (Table 3, Figs. 3, 4a–4c)

Dorsal anchors with guard length 4 to 5 times the shaft length. U-shaped indentation at the base. Point short and curved (e = 11 μm). Ventral anchors 10 μm smaller than dorsal ones. Blade equally long as in dorsal anchors, but base of the ventral anchors shorter, with deeper V-shaped indentation. Hooks pair I long (>1.7 times the length of hooks pair II [35]), with rectangular-shaped shaft, slightly broader than the anterior, larval part. Hooks pair III–VII short (<2 times the length of hooks pair II [35]). Ventral transverse bar large (X = 53 μm) and broad (W = 9 μm) with lateral extension on each arm. Extension long, covers around 70% of the length of each arm, except for the proximal and distal ends. Dorsal transverse bar with short auricles (h = 12 μm), with usual morphology for species of *Cichlidogyrus* infecting species of *Tylochromis* (i.e. auricles in continuity with dorsal bar anterior face [23, 28]). Penis, thick-walled, tubular with a sinuous curve most proximally, followed by a 360° loop. At the distal end, the penis curves approximately 60°. Basal bulb granulated. Heel absent. Accessory piece not directly connected to the basal bulb. It starts at the height of the loop, where it is club-shaped and ridged. Further distally, it narrows until it is thinner than the penis.

**Figure 3.**
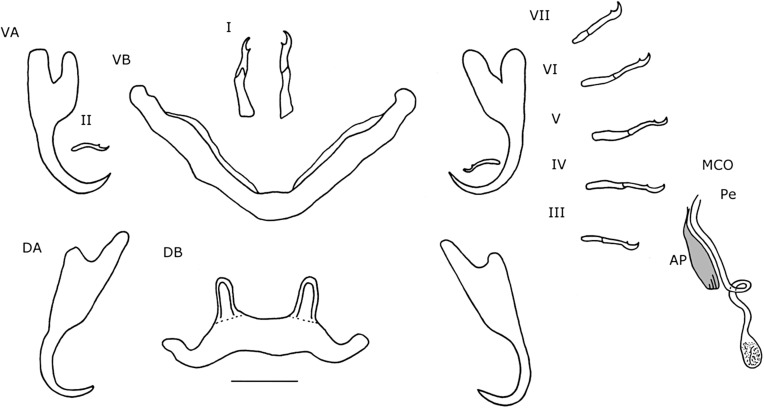
Haptoral and genital hard parts of *Cichlidogyrus bixlerzavalai* n. sp. from *Tylochromis praecox*. Accessory piece of the MCO in grey, to highlight the plate-like structure of the accessory piece. I–VII, hooks; AP, accessory piece; DA, dorsal anchors; DB, dorsal transverse bar; MCO, male copulatory organ; Pe, penis; VA, ventral anchors; VB, ventral transverse bar. Scale bar: 20 μm.

**Figure 4.**
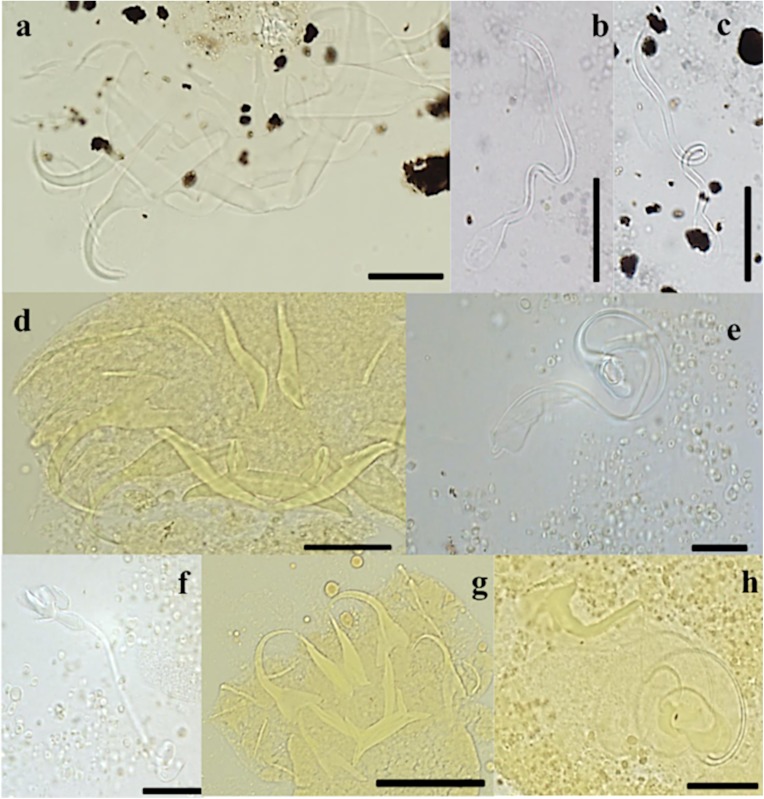
Compound micrographs of (a–c) *Cichlidogyrus bixlerzavalai* n. sp. from *Tylochromis praecox*: haptor of holotype (a), MCO of holotype (b) and MCO of paratype (c); of (d–f) *Cichlidogyrus calycinus* n. sp. from *Hemichromis elongatus*: haptor of holotype (d), MCO of holotype (e), vagina of holotype (f); of (g–h) *Cichlidogyrus polyenso* n. sp. from *Hemichromis elongatus*: haptor (g), MCO (h). Scale bar 20 μm, except for (g) 50 μm.

**Table 3.** Measurements of *Cichlidogyrus bixlerzavalai* n. sp., *C. omari* n. sp. and *C. reversati*. Note the size difference in dorsal bar length, x between the two groups of *C. reversati*. All measurements in μm as the average ± standard deviation, count and range (in parentheses).

Species	*C. bixlerzavalai* n. sp.	*C. omari* n. sp.	*C. reversati*	*C. reversati*
Host	*T. praecox*	*T*. *praecox*	*C. tholloni*	*P. cabrae*
Locality	2	2	8	Mouth of Bas Koilou River, ROC
Reference	Present study	Present study	Present study	Pariselle and Euzet [33]
Number of specimens	4	1	8	30
Ventral anchor				
Total length, a	40, 3 (39–41)	32	43, 8 (41–45)	44 ± 2 (40–49)
Blade length, b	39, 3 (35–41)	26	42, 8 (40–44)	44 ± 1.6 (40–46)
Shaft length, c	2, 3 (1–3)	4	4, 8 (3–7)	3 ± 1.3 (1–9)
Guard length, d	13, 3 (11–14)	12	16, 8 (13–17)	11 ± 1.5 (6–16)
Point length, e	11, 3 (9–12)	7	17, 8 (16–19)	16 ± 1.3 (13–19)
Dorsal anchor				
Total length, a	50, 3 (49–51)	31	57, 8 (55–60)	59 ± 1.9 (53–63)
Blade length, b	38, 3 (37–39)	22	40, 8 (37–43)	42 ± 1.8 (36–45)
Shaft length, c	4, 3 (3–5)	6	4, 8 (2–6)	4 ± 1.8 (2–14)
Guard length, d	22, 3 (18–24)	15	28, 8 (25–30)	21 ± 1.7 (17–28)
Point length, e	11, 3 (10–11)	8	14, 8 (13–16)	14 ± 1.1 (11–16)
Uncinuli				
Length, I	24, 3 (21–26)	14	25, 7 (23–29)	25 ± 1.5 (22–28)
Length, II	12, 3 (10–13)	10	12, 5 (11–13)	12 ± 0.8 (9–14)
Length, III	19, 3 (17–21)	14	20, 8 (15–22)	18 ± 0.8 (17–21)
Length, IV	21, 3 (21–22)	19	23, 8 (20–27)	22 ± 0.9 (20–25)
Length, V	21, 3 (21–22)	23	25, 8 (23–28)	24 ± 1.3 (20–27)
Length, VI	21, 3 (20–22)	18	22, 7 (20–24)	21 ± 0.8 (20–23)
Length, VII	19, 3 (16–22)	17	19, 6 (18–23)	19 ± 0.9 (15–20)
Ventral bar				
Branch length, X	53, 3 (48–57)	39	48, 7 (35–55)	45 ± 3.2 (37–50)
Maximum width, W	9, 3 (8–10)	5	8, 7 (7–11)	9 ± 1.4 (6–13)
Dorsal bar				
Total, length, x	59, 3 (57–61)	62	57, 7 (53–64)	46 ± 2.3 (42–51)
Maximum width, w	9, 3 (8–10)	6	10, 7 (8–12)	11 ± 1.4 (9–15)
Distance between auricles, y	17, 3 (15–18)	8	20, 7 (13–24)	15 ± 2.4 (12–24)
Auricle length, h	12, 3 (9–15)	11	20, 7 (17–23)	18 ± 2.2 (14–24)
MCO				
Penis length, Pe	65, 3 (61–70)	48	27, 8 (24–31)	33 ± 1.4 (31–36)
Length of accessory piece, AP	26, 2 (22–32)	58	32, 8 (28–34)	/
Heel length, He	N.A.	2	N.A.	N.A.
Length of accessory piece straight, Aps			21, 8 (16–26)	20 ± 1.5 (17–23)
Total body length	449, 3 (424–488)	460	364, 8 (312–429)	713 ± 106 (545–1011)

#### Remarks

Species of *Cichlidogyrus* that infect species of *Tylochromis* have reduced auricles on the dorsal transverse bar. The accessory piece does not connect to the basal bulb of the penis [23, 28]. However, a thin, filamentous connection was described for *C. sigmocirrus* Pariselle, Bitja Nyom, Bilong Bilong, 2014 and *C. chrysopiformis* Pariselle, Bitja Nyom, Bilong Bilong, 2014 [36], but with uncertainty. Both species were described from *Tylochromis sudanensis* Daget, 1954 from the Cross River in Cameroon [36]. Furthermore, all species have a tubular curved penis, referred to as a spirally-winding penis [23]. *Cichlidogyrus bixlerzavalai* n. sp. resembles *C. muzumanii* Muterezi Bukinga, Vanhove, Van Steenberge & Pariselle, 2012 the most, as both species have large anchors with a long guard, elongated hooks pair I and a curved tubular penis with an accessory piece at the distal end of the penis that runs to almost half of the length of the penis. The major difference between these species is that in *C. muzumanii*, the accessory piece engulfs the penis partially, while in *C. bixlerzavalai* n. sp., the accessory piece lies separate from the penis. This has never been observed for species of *Cichlidogyrus*. It remains unclear if and where the accessory piece attaches to the penis in *C. bixlerzavalai* n. sp. However, it is certain that the accessory piece does not connect to the basal bulb proximally and that the accessory piece does not engulf the distal end of the penis. One specimen has a bulge-shaped extension at the distal end of the penis, but this is probably an artefact, because it is an open structure, clearly observable and absent on other specimens. Additionally, the specimen with the extension is also the only one of which the distal end of the penis is oriented towards the accessory piece. It is observed on this specimen that at the 360° loop the penis turns in the other direction. However, this directional change is likely the result of the flattening of the MCO. On one specimen, ridges at the proximal end of the accessory piece were not observed.

### *Cichlidogyrus calycinus* Kusters, Jorissen, Pariselle & Vanhove n. sp.

urn:lsid:zoobank.org:act:D4922EBE-7FBE-425C-BBF0-C90E39DB7171

Type host: *Hemichromis elongatus* (Guichenot, 1861).

Infection site: Gills.

Type locality: Pond near Kila Kindinga 5°29′7″S 14°53′3.8″E.

Other localities: Mvuazi River (Lower Congo) 5°19′S 15°7′E.

Material: Fifteen whole-mounted specimens of which six are fixed in Hoyer’s medium (including the holotype), the other nine in Malmberg’s solution.

Holotype: M.T. 38316.

Paratypes: M.T. 38312–13, 38317–18, 38321, 38326, 38329, 38331, 38333, 38335, KN10046–47, https://laji.fi/en/view?uri=luomus:KN.10046 and https://laji.fi/en/view?uri=luomus:KN.10047, SAMC-A090066, NHMUK 2018.1.31.1.

Symbiotype: RMCA_Vert_2015.030.P.0020.

Paratype host vouchers: RMCA_Vert_2015.030.P.0019, RMCA_Vert_2015.030.P.0021, RMCA_Vert_2015.030.P.0022.

Etymology: The species epithet in Latin refers to the cup-shaped distal end of the vagina (calyx, Latin: cup) and is an adjective.

Authorship: Note that the authors of the new taxon are different from the authors of this paper; Article 50.1 and Recommendation 50A of International Code of Zoological Nomenclature [13].

#### Description (Table 4, Figs. 4d–4f, 5)

Dorsal anchors, with long guard (d = 17 μm), which is more than twice the shaft length (d = 7 μm). Blade long and curved. Ventral anchors on average 4 μm smaller than dorsal anchors, but with longer point. Hooks I and V long. Hooks III–IV and VII–VII sometimes long, sometimes short. Ventral transverse bar with small extension at distal third of each arm. Dorsal transverse bar slightly concave, with developed, but rather short auricles (h = 15 μm). Penis long, thin, tubular and makes a long turn of almost 360° after leaving the basal bulb and ends near the middle of the distal plate of the accessory piece. Heel oval. Accessory piece proximally broader than penis and with bean-shaped extension at one sixth of the length, where accessory piece and penis meet. Further distally, the accessory piece narrows to the width of the penis and follows its trajectory, but ends further distally. Accessory piece ends distally in an irregularly-shaped plate. Distal plate with separate elongated structure in the middle. The plate itself is twice as long as it is broad and with rounded edges. Elongated structure longer than plate and reaches further proximally than plate. Proximal from where the elongation overshoots the plate, it touches the penis and narrow part of the accessory piece. Vagina large and tubular. Distally, the vagina has a forceps-shaped structure, engulfed by a semi-hollow structure, which resembles the cup of a flower. More proximally, the forceps-like structure thickens after which it narrows into a long and narrow tube. At the proximal end, the vagina makes a loop and broadens slightly.

**Figure 5.**
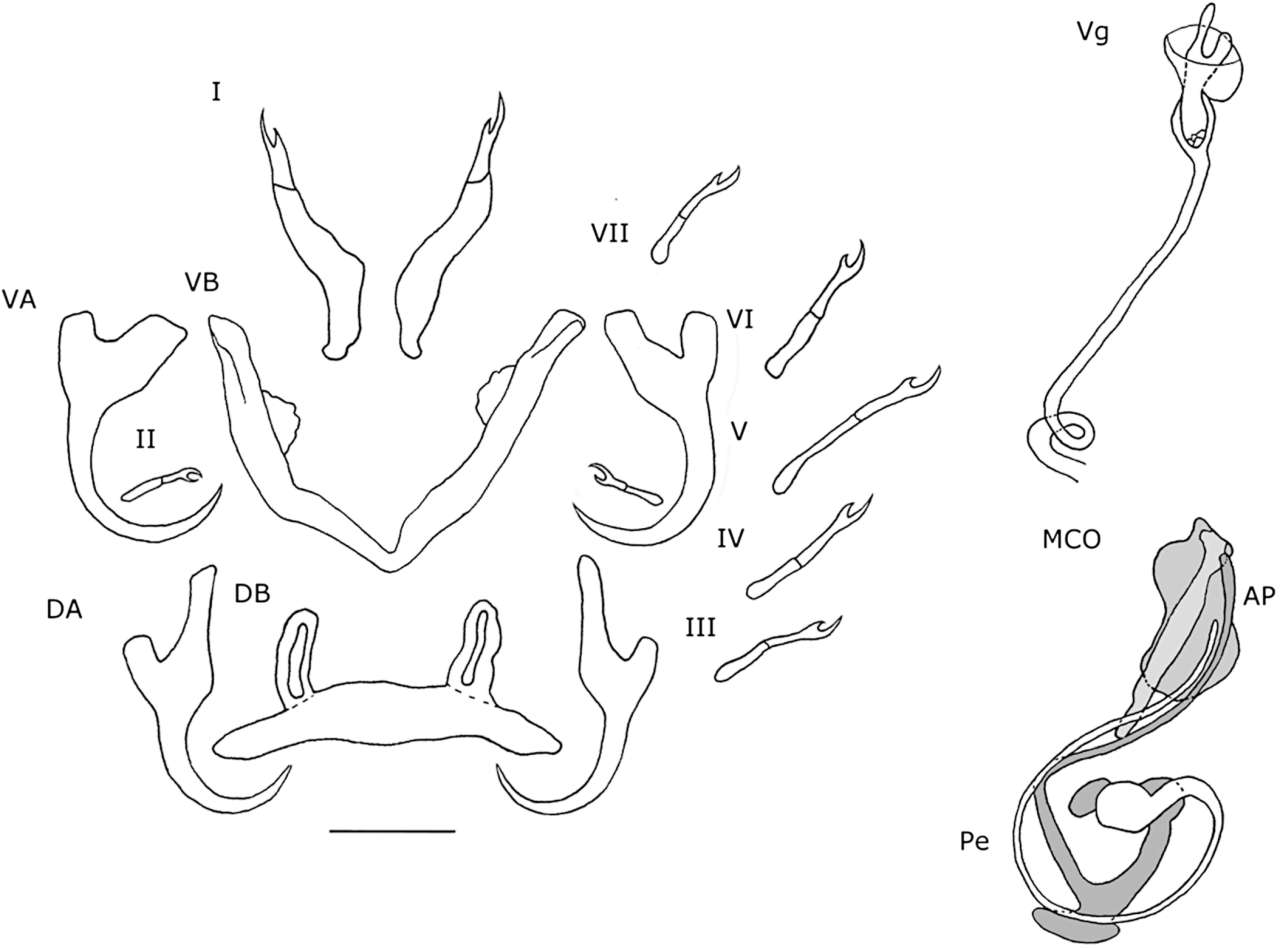
Haptoral and genital hard parts of *Cichlidogyrus calycinus* n. sp. from *Hemichromis elongatus.* Accessory piece of the MCO in grey, to highlight the plate-like structure of the accessory piece. I–VII, hooks; AP, accessory piece; DA, dorsal anchors; DB, dorsal transverse bar; MCO, male copulatory organ; Pe, penis; VA, ventral anchors; VB, ventral transverse bar; Vg, vagina. Scale bar: 20 μm.

**Table 4.** Measurements of *Cichlidogyrus calycinus* n. sp., *C. teugelsi*, *C. polyenso* n. sp., *C. euzeti*, *C. kmentovae* n. sp., *C.* cf. *bychowskii* and *C. dracolemma*. All measurements in μm as the average ± standard deviation, count and range (in parentheses).

Species	*C. calycinus* n. sp.	*C. teugelsi*	*C. polyenso* n. sp.	*C. euzeti*	*C. kmentovae* n. sp.	*C.* cf. *bychowskii*	*C. dracolemma*
Host	*H. elongatus*	*H. fasciatus*	*H. elongatus*	*H. fasciatus*	*H. stellifer*	*H. bimaculatus*	*H. letourneuxi*
Locality	4 & 6	Kounougou River, Ivory Coast	4	Benin & Cameroon	7	Congo River at Bokalaka, DRC	Niokolo Koba River,
Reference	Present study	Pariselle and Euzet [34]	Present study	Dossou and Birgi [6]	Present study	Messu Mandeng et al. [22]	Řehulková et al. [41]
Number of specimens	15	30	6	30	6	5	1 (Holotype)
Ventral anchor							
Total length, a	35, 7 (30–38)	36 ± 1.1 (33–38)	40, 4 (37–43)	40–45	32, 4 (30–35)	34 (31–37)	25
Blade length, b	30, 7 (26–33)	31 ± 1.4 (29–34)	38, 4 (37–39)	30–35	27, 3 (24–29)	28 (26–31)	24
Shaft length, c	7, 7 (4–12)	5 ± 1 (3–7)	4, 4 (2–5)	8–10	4, 4 (2–5)	5 (4–9)	3
Guard length, d	11, 7 (9–14)	11 ± 1.5 (8–14)	15, 4 (14–16)	10–12	13, 4 (12–15)	10 (9–14)	8
Point length, e	13, 7 (9–17)	15 ± 1.2 (12–17)	15, 4 (13–19)	10–12	12, 4 (10–15)	12 (10–13)	9
Dorsal anchor							
Total length, a	39, 7 (33–43)	41 ± 1.6 (35–45)	46, 4 (43–47)	40–50	32, 5 (31–34)	33 (31–35)	23
Blade length, b	26, 7 (22–28)	27 ± 1.2(25–31)	31, 4 (29–33)	28–30	22, 5 (19–23)	23 (21–25)	17
Shaft length, c	7, 6 (5–10)	5 ± 0.9 (3–7)	5, 4 (2–7)	8–10	3, 5 (2–4)	5 (4–7)	2
Guard length, d	17, 6 (15–20)	17 ± 1.7 (12–20)	22, 4 (20–23)	20–25	16, 5 (15–18)	13 (10–15)	12
Point length, e	10, 7 (9–12)	11 ± 0.7 (10–13)	9, 4 (7–10)	8–10	9, 5 (9–10)	9 (6–10)	6
Uncinuli							
Length, I	32, 7 (22–42)	31 ± 1.6 (28–37)	43, 4 (38–47)	35–40	32, 4 (29–34)	34 (33–37)	/
Length, II	9, 2 (8–9)	10 ± 0.5 (9–12)	10, 3 (9–11)	10	10, 4 (10–11)	11 (11–11)	11
Length, III	17, 5 (13–21)	18 ± 1.2 (17–23)	21, 4 (19–22)	15–20	20, 4 (19–22)	21 (19–22)	17
Length, IV	19, 5 (15–23)	20 ± 1.5 (18–25)	22, 4 (20–23)	15–20	23, 4 (22–26)	22 (20–23)	16
Length, V	25, 6 (21–31)	24 ± 1.2 (21–29)	24, 4 (22–27)	15–20	25, 4 (21–27)	25 (23–27)	17
Length, VI	20, 6 (16–23)	21 ± 1.1 (19–23)	25, 4 (20–28)	15–20	21, 4 (18–23)	24 (22–25)	18
Length, VII	17, 6 (13–21)	18 ± 0.8 (16–20)	22, 4 (21–25)	15–20	20, 4 (19–22)	22 (20–23)	16
Ventral bar							
Branch length, X	34, 6 (26–38)	34 ± 2.7 (31–39)	46, 3 (40–54)	40	31, 4 (28–34)	31 (29–36)	18
Maximum width, W	5, 6 (3–6)	6 ± 0.5 (4–7)	6, 4 (5–7)	/	5, 4 (4–5)	4 (3–5)	3
Dorsal bar							
Total, length, x	40, 6 (31–55)	35 ± 1.7 (32–38)	50, 3 (41–58)	45	37, 5 (34–43)	36 (31–40)	22
Maximum width, w	7, 6 (5–9)	8 ± 0.9 (7–10)	8, 4 (6–11)	/	7, 5 (6–8)	7 (6–8)	5
Distance between auricles, y	15, 6 (12–25)	12 ± 1 (10–13)	23, 3 (20–27)	20	12, 5 (11–16)	12 (9–14)	10
Auricle length, h	10, 6 (9–15)	11 ± 1.2 (8–14)	12, 4 (11–13)	10–12	9, 5 (8–11)	9 (8–11)	5
MCO							
Penis length, Pe	104, 6 (77–119)	99 ± 4.3 (90–111)	256, 5 (225–284)	375–390	137, 5 (103–187)	185 (108–203)	95
Length of accessory piece, AP	52, 7 (36–63)	74 ± 3.9 (69–87)	127, 5 (112–163)	130	77, 4 (65–88)	185 (108–203)	51
Heel length, He	14, 5 (4–24)	/	4, 5 (3–5)	/	3, 5 (2–4)	3 (2–5)	/
Vagina							
Vagina length, L	85, 7 (67–105)	72 ± 7.2 (61–88)	129	/	29	N.A.	46
vagina width, l	4, 7 (3–6)	3 ± 0.3 (3–4)	/	/	/	/	/
Total body length	/	528 ± 55.9 (403–605)	497, 5 (436–583)	600–700	/	/	/

#### Remarks

In general, the morphology of the haptor of species of *Cichlidogyrus* that infect species of *Hemichromis* hardly varies [22, 34]. Thus, the morphology and measurements of the haptoral sclerites of *C. calycinus* n. sp. are nearly identical to that of *C. teugelsi* Pariselle & Euzet, 2004. The main distinction in these structures is that the dorsal bar of *C. calycinus* n. sp. is larger (x = 40 μm, 31–55 μm, n = 15) than that of *C. teugelsi* (x = 35 μm, 32–38 μm, n = 30). The major morphological differences between the two are found on the MCO and vagina. Both have a long tubular penis that almost makes a 360° turn and an accessory piece with a bean-shaped extension at 1/6 of its length. Both species differ at the distal end of the accessory piece. In *C. teugelsi*, at the distal end the accessory piece makes a loop, while in *C. calycinus* n. sp. it forms an irregularly-shaped plate with an elongated structure attached to it. Furthermore, the narrow part of the accessory piece in *C. calycinus* n. sp. reaches further distally than that in *C. teugelsi*, which broadens and forms the loop structure at 5/6 of the length of the penis. Also, the accessory piece is on average 22 μm longer in *C. teugelsi* than in *C. calycinus* n. sp., while the penes differ 5 μm in length, with *C. calycinus* n. sp. having the longer one (Pe = 104 μm). The heel of *C. teugelsi* is irregularly shaped, whilst that of *C. calycinus* n. sp. is oval shaped. In some specimens of *C. calycinus* n. sp., the heel seemed longer. The vaginae of both species are also similar in morphology and size as they are long, tubular structures that make a loop at their proximal end. However, the distal ends are different. In *C. teugelsi* the distal end is a slightly sclerotized plate, while in *C. calycinus* n. sp. there is a forceps-like structure and an overall cup shape.

### *Cichlidogyrus polyenso* Jorissen, Pariselle & Vanhove n. sp.

urn:lsid:zoobank.org:act:C1165346-CFFB-45C7-951E-0D3E094084B9

Type host: *Hemichromis elongatus* (Guichenot, 1861).

Infection site: Gills.

Type locality: Pond near Kila Kindinga 5°29′7″S 14°53′3.8″E.

Material: four whole-mounted specimens in Malmberg’s solution (including the holotype) and two in Hoyer’s medium.

Holotype: M.T. 38330.

Paratypes: M.T. 38314–15, 38332, NHMUK 2018.1.31.3, KN10050 https://laji.fi/en/view?uri=luomus:KN.10050.

Symbiotype: RMCA_Vert_2015.030.P.0021.

Paratype host vouchers: RMCA_Vert_2015.030.P.0019, RMCA_Vert_2015.030.P.0020.

Etymology: Species epithet is derived from Zen Buddhism and the Japanese language where an *enso* is a hand-drawn circle. This refers to the shape of the penis, as it is coiled. Species epithet consists of a prefix and a noun.

Authorship: Note that the authors of the new taxon are different from the authors of this paper; Article 50.1 and Recommendation 50A of International Code of Zoological Nomenclature [13].

#### Description (Table 4, Figs. 4g–4h, 6)

Dorsal anchors with guard length four to five times the shaft length. Guard elongated and narrow. Point short (e = 9 μm). Ventral anchors 6 μm smaller than dorsal ones. Base with shallow, but wide indentation. Point long (e = 15 μm). Hooks pair I elongated. Secondary shaft (see [35]) widens gradually and ends rounded. Hooks III-VII short. Dorsal transverse bar slightly concave with narrow auricles that are far apart (y = 23 μm). Ventral transverse bar V-shaped with small extension at 1/3 of the distal ends of each arm. MCO with long tubular penis, which is spirally coiled and makes two to three loops. Distally from the loops, the penis makes a 90° turn and continues straight before ending. Short, rectangular heel with rounded edges (He = 4 μm). Accessory piece attaches to basal bulb, proximally broad but narrows distally of the basal bulb. Accessory piece makes two to three loops within the loops of the penis. Distally from the loops, the accessory piece leaves the space within the loops of the penis, turns 120°, meets the penis again and forms a plate resembling the tail of cetaceans, but with asymmetrical lateral ends and sides. At the proximal side, closest to the basal bulb, the plate is convex and results in a sharp point. The distal side is partially concave and partially convex and ends in a hook-like structure. The penis overshoots the plate and ends further distally. The vagina is large, tubular. Most proximally it corkscrews once, after which it makes a short loop. Soon after this it turns 180° to stack two loops of tube after which it ends distally, shortly after leaving the coil.

**Figure 6.**
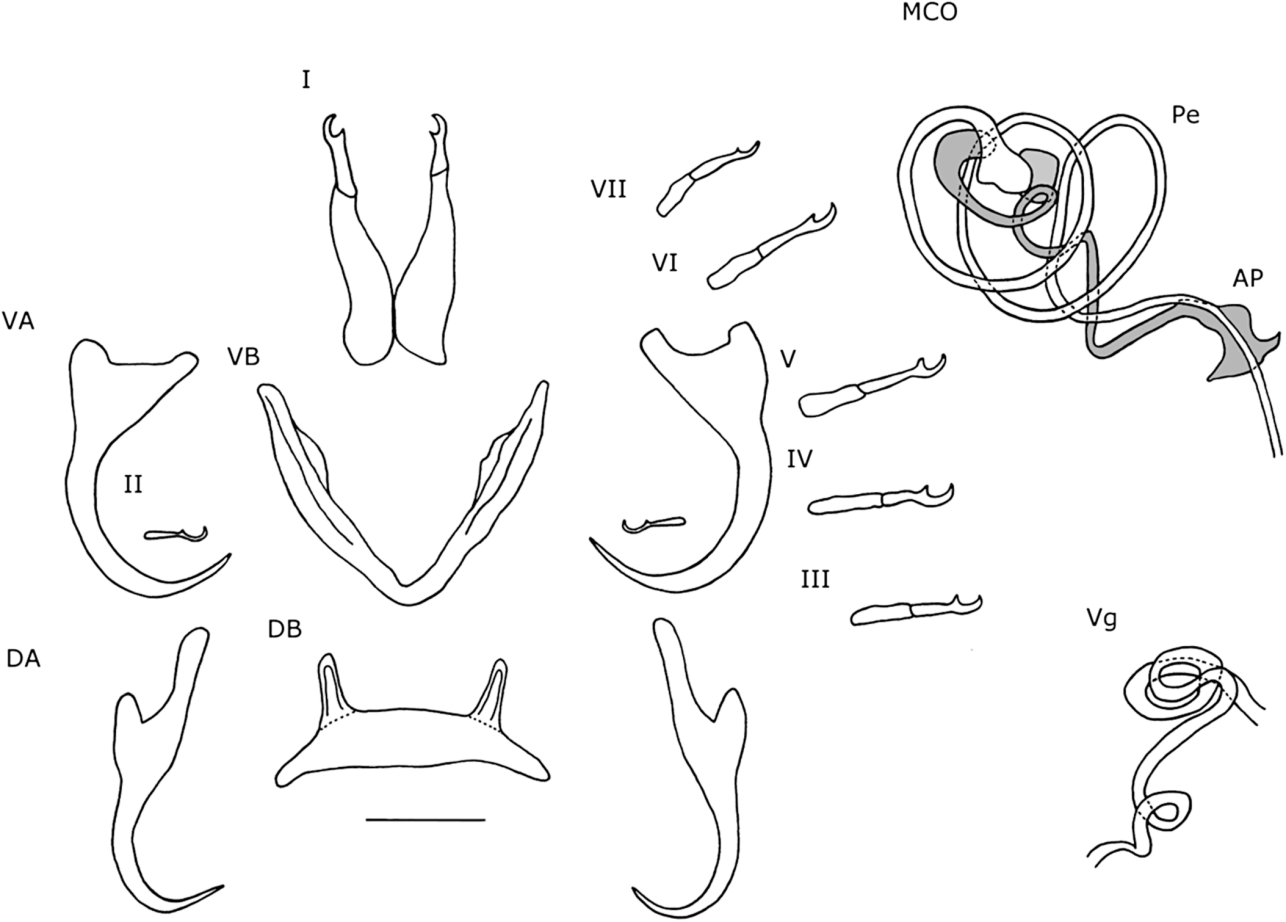
Haptoral and genital hard parts of *Cichlidogyrus polyenso* n. sp. from *Hemichromis elongatus.* Accessory piece of the MCO in grey, to highlight the plate-like structure of the accessory piece. I–VII, hooks; AP, accessory piece; DA, dorsal anchors; DB, dorsal transverse bar; MCO, male copulatory organ; Pe, penis; VA, ventral anchors; VB, ventral transverse bar; Vg, vagina. Scale bar: 20 μm.

#### Remarks

*Cichlidogyrus polyenso* n. sp. has multiple characteristics in common with *C. euzeti* Dossou & Birgi, 1984. Similarities are that both species have elongated hooks pair I, short hooks pairs III-VII, dorsal anchors with a long guard and short point, ventral anchors with a long point, a spirally-coiled penis, accessory piece and vagina. The major difference with *C. euzeti* is that the penis of *C. polyenso* n. sp. has fewer coils, two to three, rather than the four to five in *C. euzeti*, and is shorter in length. Moreover, the accessory piece is less coiled. Additionally, the vagina in *C. polyenso* n. sp. is more compacted, because it coils, while the vagina of *C. euzeti* is a long tube that winds, but never coils. Also, *C. polyenso* n. sp. has slightly larger ventral anchors (b = 38 μm, e = 15 μm) and hooks (I = 43 μm, V = 24 μm) than does *C. euzeti* (b = 30–35 μm, e = 10–12 μm, I = 35–40 μm, V = 15–20 μm). *Cichlidogyrus euzeti* is reported from Benin and Cameroon [6, 22] and the ROC [35]. Other species that have a similar sclerite morphology are *C. longicirrus* Paperna, 1965 and *C. sanseoi* Pariselle & Euzet, 2004, both from *H. fasciatus* Peters, 1858 and the former from *Chromidotilapia guentheri* (Sauvage, 1882) as well, but both parasites have a longer MCO with more coils than do *C. polyenso* n. sp. and *C. euzeti*.

### *Cichlidogyrus kmentovae* Jorissen, Pariselle & Vanhove n. sp.

urn:lsid:zoobank.org:act:8A9E340B-2B30-428E-B994-AB9E296F5DEC

Type host: *Hemichromis stellifer* Loiselle, 1979.

Infection site: Gills.

Type locality: Mbola River near Tshianya Village 05°52′09.8″S 12°39′52.6″E.

Material: Six whole-mounted specimens in Malmberg’s solution.

Holotype: M.T. 38338.

Paratypes: M.T. 38336–37, NHMUK 2018.1.31.5, SAMC-A090068.

Symbiotype: AB53952197.

Etymology: Species epithet refers to biologist Nikol Kmentová (Czech Republic), an enthusiastic researcher on the monogenean fauna of Lake Tanganyika and is a noun (name) in the genitive case.

Authorship: Note that the authors of the new taxon are different from the authors of this paper; Article 50.1 and Recommendation 50A of International Code of Zoological Nomenclature [13].

#### Description (Table 4, Figs. 7, 8a–8b)

Dorsal anchors with elongated guard that is four to five times the shaft length. Indentation, smooth, convex up to the shaft. Short point (e = 9 μm). Ventral anchors of same size (a = 32 μm) but with a more robust blade and base. Guard shorter (d = 13 μm). Indentation at the base less convex than in dorsal anchors. Hooks pair I large with broad primary shaft and even broader secondary shafts. Hooks pair IV–V long. Hooks pair VII short. Hooks VI of variable size, some long, some short. Pair III short and sometimes differs less than 0.2 μm from twice the length of II. Dorsal transverse bar slightly concave with short extensions. Auricles developed, but short (h = 9 μm). Ventral transverse bar small (X = 31 μm) and narrow (W = 5 μm) with extension that starts halfway along the course of each arm and reaches almost to the distal tip. MCO large with a long, thin, tubular penis (Pe = 137 μm), which gradually narrows over its course and makes a large turn of 270° at about the middle of its length. More distally, the penis turns 90° and meets the accessory piece. Basal bulb with small rectangular heel with rounded edges (He = 3 μm). Accessory piece departs from the basal bulb and makes a short 180° turn after which it broadens and meets the penis. At the distal end, the accessory piece connects to a semi-circular plate with seven discernible grooves on its surface. At the junction between the semi-circular plate and the rest, two small, sharp extensions protrude towards the semi-circular plate. Vagina consists of a thick-walled bulbous structure connected to a tube.

**Figure 7.**
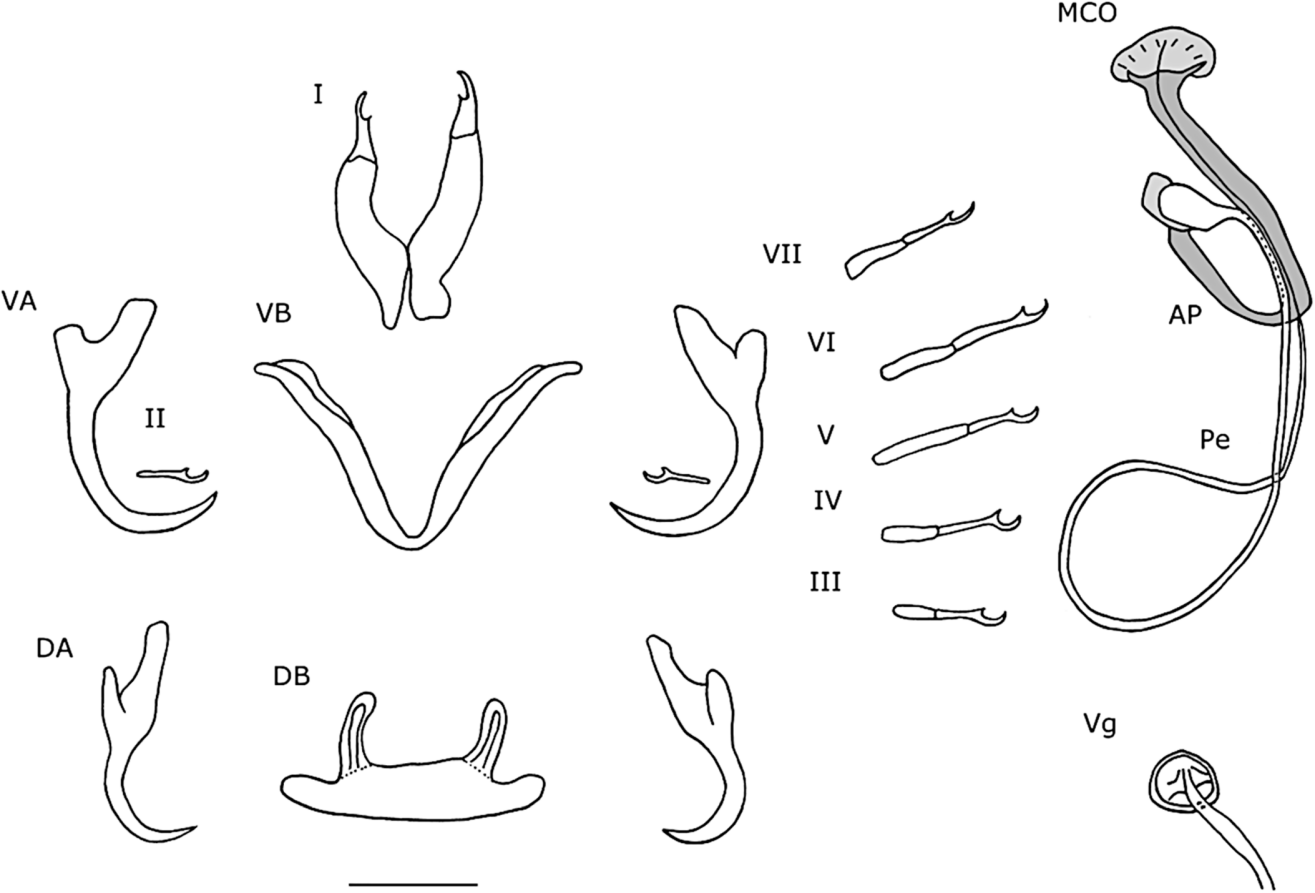
Haptoral and genital hard parts of *Cichlidogyrus kmentovae* n. sp. from *Hemichromis stellifer.* Accessory piece of the MCO in grey, to highlight the plate-like structure of the accessory piece. I-VII, hooks; AP, accessory piece; DA, dorsal anchors; DB, dorsal transverse bar; MCO, male copulatory organ; Pe, penis; VA, ventral anchors; VB, ventral transverse bar; Vg, vagina. Scale bar: 20 μm.

**Figure 8.**
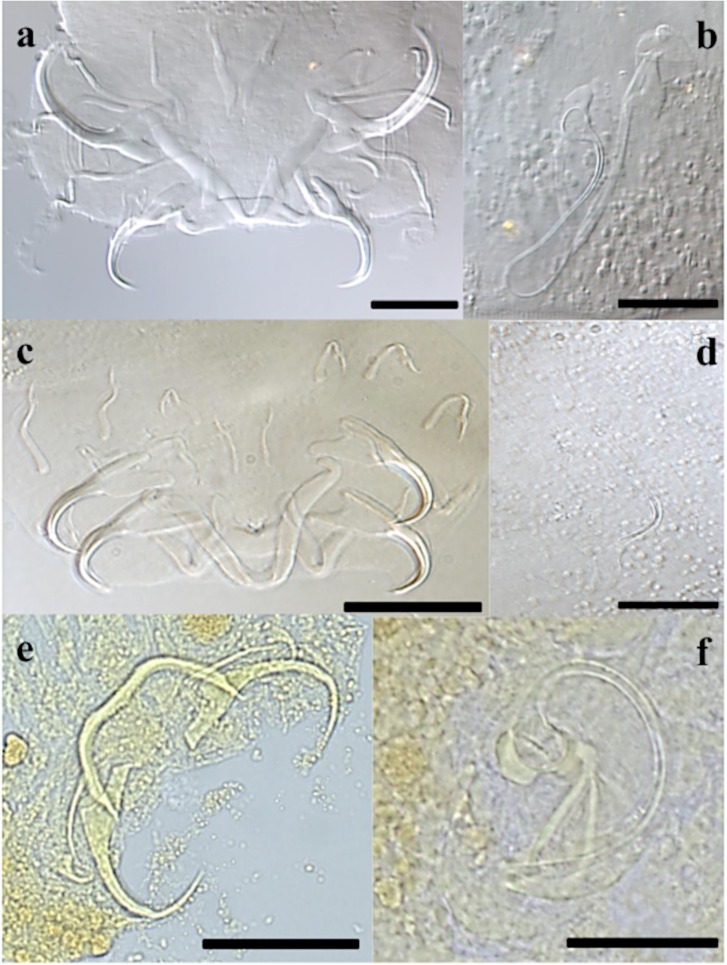
DIC micrographs of (a–b) *Cichlidogyrus kmentovae* n. sp. from *Hemichromis stellifer*: haptor (a), MCO (b); of (c–d) *Cichlidogyrus omari* n. sp. from *Tylochromis praecox*: haptor of holotype (c), MCO of holotype (d); compound micrographs of (e–f) *Onchobdella ximenae* n. sp. from *Hemichromis elongatus*: haptor (e), MCO (f). Scale bar 20 μm, except for (e) 50 μm.

#### Remarks

*Cichlidogyrus kmentovae* n. sp. closely resembles *C. amieti* Doussou & Birgi, 1983; *C. bychowskii sensu* Paperna, 1965 and *C. dracolemma* Řehulková, Mendlová & Šimková, 2013. *Cichlidogyrus bychowskii* was originally described by Markevitch, 1934 [17] as *Ancyrocephalus bychowskii* from the Leningrad aquarium from *Hemichromis bimaculatus* Gill, 1862. No type material was deposited and a drawing of the MCO and vagina was lacking [17]. However, the penis was described as a long tube that was coiled many times [17]. A redescription of *C. bychowskii* on new material from Southern Ghana from the same host species followed in 1965 by Paperna [25], this time with a drawing of all sclerotized structures. However, the penis was represented as a long tube that makes one large loop instead of multiple coils. Paperna did not address the difference in morphology of the penis and based his diagnosis on the total length of the animal and the morphology of some haptoral sclerites [25, 41]. Therefore, Messu Mandeng et al., Řehulková et al. [22, 41] and the present authors suspect that Paperna misidentified his specimens, which are different from *C. bychowskii* and represent a new species. Furthermore, because the description of Markevitch [17] is not sufficient to recognize the animal and fails to illustrate its key characteristics, *Cichlidogyrus bychowskii* is considered a nomen dubium. It was previously considered a nomen nudum [22], but the term was misused. In Messu Mandeng et al. [22] specimens were found that resembled the species of Paperna, 1965 and were considered as *C.* cf. *bychowskii*. The present authors will use *C.* cf. *bychowskii* for the specimens described by Paperna, 1965 and collected by Messu Mandeng et al. [22] and stress that the species would need a new name in time, preferably, based on a description of new material. No voucher material was deposited by Paperna [25]. The difference between *C. kmentovae* n. sp. (L = 29 μm) and *C. amieti* is that the vagina of *C. amieti* is longer (L = 65–70 μm from [5]) and the distal plate of the accessory piece is absent. *Cichlidogyrus dracolemma* and *C.* cf. *bychowskii* both have a distal plate, but the vagina of *C. dracolemma* is longer (L = 46 μm) and thicker than in *C. kmentovae* n. sp. (L = 29 μm). Both *C. dracolemma* and *C.* cf. *bychowskii* also have a broadened, irregularly-shaped distal plate with a large hook. In *C. kmentovae* n. sp. the distal plate is semi-circular and has grooves. *Cichlidogyrus* cf. *bychowskii* and *C. kmentovae* n. sp. both have a vagina with a circular, broadened proximal part, from which a thin, tubular structure extends to the genital pore, but in *C.* cf. *bychowskii*, this tube makes a 360° loop. With regard to the length of the hooks, there was a categorical size difference in length between pairs III–VII, which is not mentioned in the descriptions of other species. However, the average length of pairs III and VI were short, whilst pairs IV–VI were long for *C.* cf. *bychowskii* [22]. In *C. kmentovae* n. sp., some measurements of hook pair III differed less than 0.2 μm from twice the length of pair II. It is, therefore, not always straightforward to categorize the length of the hooks. Our sample size was limited, and thus a larger dataset of measurements should further clarify the discrepancy in hook lengths.

### *Cichlidogyrus omari* Jorissen, Pariselle & Vanhove n. sp.

urn:lsid:zoobank.org:act:73B533F8-2E50-4A01-BF82-80DF705281CB

Type host: *Tylochromis praecox* Stiassny, 1989.

Infection site: Gills.

Type locality: Muila Kaku, mangroves near Lower Congo River 05°59′33″S 12°35′03.2″E.

Material: One whole-mounted specimen fixed in Malmberg’s solution.

Holotype: M.T. 38342.

Etymology: The species epithet is a homage to lead guitarist Omar Rodriguez-Lόpez from bands At the drive-in, The Mars Volta, Antemasque, The Omar Rodriguez-Lopez group and Bosnian Rainbows and is a noun (name) in the genitive case.

Authorship: Note that the authors of the new taxon are different from the authors of this paper; Article 50.1 and Recommendation 50A of International Code of Zoological Nomenclature [13].

#### Description (Table 3, Figs. 8c–8d, 9)

Dorsal anchors small (a = 31 μm) with a short point (e = 7 μm). Ventral anchors of similar size (a = 32 μm) with a slightly longer point (e = 8 μm) and a slightly bulkier base. All hooks are small. Ventral transverse bar small (X = 39 μm), thin (W = 5 μm) and V-shaped. Extension absent. Dorsal transverse bar with short, stubby auricles (h = 11 μm). Penis has a rectangular basal bulb with rounded edges and a small rectangular heel with rounded edges. Penis is thin, tubular and curved. Accessory piece lies distally of the penis and consists of two parts, the most proximal one being a mantle-like structure that engulfs the penis at the height of the curvature and further distally from there. This mantle-like structure ends proximally in a point. Distally, it is connected to a second part, which is an elongated, blunt structure with rounded edges.

**Figure 9.**
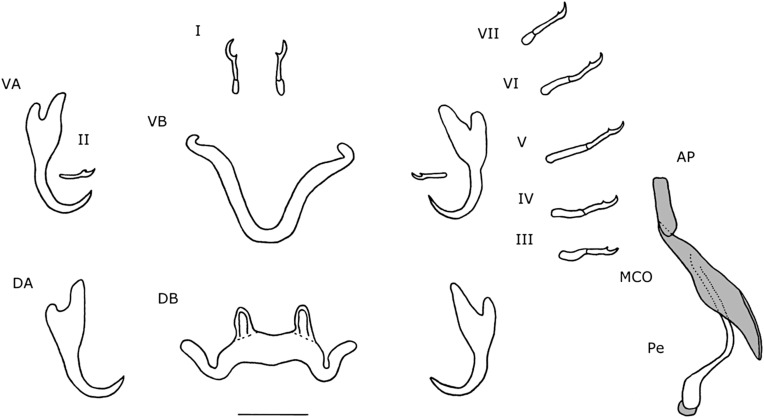
Haptoral and genital hard parts of *Cichlidogyrus omari* n. sp. from *Tylochromis praecox*. Accessory piece of the MCO in grey, to highlight the plate-like structure of the accessory piece. I–VII, hooks; AP, accessory piece; DA, dorsal anchors; DB, dorsal transverse bar; MCO, male copulatory organ; Pe, penis; VA, ventral anchors; VB, ventral transverse bar. Scale bar: 20 μm.

#### Remarks

*Cichlidogyrus omari* n. sp. has a small, rectangular heel, an accessory piece divided into two parts, and small transversal bars, anchors and hooks. These features have not been seen until now for species of *Cichlidogyrus* infecting *Tylochromis* spp. However, the accessory piece is large, mantle-shaped and partially engulfs the penis, which is also seen in *C. kothiasi* Pariselle & Euzet, 1994; *C. djietoi* Pariselle, Bitja Nyom & Bilong Bilong, 2014; *C. chrysopiformis* and *C. mulimbwai* Muterezi Bukinga, Vanhove, Van Steenberge and Pariselle, 2012. *Cichlidogyrus bixlerzavalai* n. sp. and C. *omari* n. sp. are easily distinguishable by the size difference in ventral transversal bar, anchors and hooks pair I. Furthermore, *C. omari* n. sp. has a penis that is curved once and it has a large accessory piece that partially engulfs the penis, whilst *C. bixlerzavalai* n. sp. has a penis that makes one loop and has a smaller accessory piece with three indentations at the distal end. It is not common practice for species of *Cichlidogyrus* to be described based on a single specimen (see *Cichlidogyrus* sp. “*T. polylepis* 3” in [36]), but on the specimen of *C. omari* n. sp. all characters are discernible and are clearly different from already described species.

### *Cichlidogyrus reversati* Pariselle & Euzet, 2003

Type host: *Tilapia cabrae* Boulenger, 1899 (now *Pelmatolapia cabrae*).

Other hosts: *Coptodon tholloni* (Poll & Thys van den Audenaerde, 1960).

Infection site: Gills.

Type locality: Mouth of Bas Kouilou River, Congo.

Other localities: Congo River at Nganda Flash station 06°02′01.8″S 12°31′48.2″E.

Material: Eight whole-mounted specimens in Malmberg’s solution.

Vouchers: M.T. 38308–09, KN10043–45 https://laji.fi/en/view?uri=luomus:KN.10043, https://laji.fi/en/view?uri=luomus:KN.10044, https://laji.fi/en/view?uri=luomus:KN.10045.

#### Remarks (Table 3, Fig. 10)

The eight recorded specimens of *C. reversati* from the Congo River had a dorsal bar length that was 9 μm longer on average and a total length of half the size compared with the specimens from the type locality. In the original drawing (see [33]), the accessory piece connected with the heel through a thin filament. However, this connection was not observed in the specimens from Lower Congo. In three specimens, an extension of the heel in another focal plane was observed and could correspond with the thin filamentous connection of the original description. Additionally, in three specimens, the distal ends of the accessory piece and the penis did not meet. Also, in all specimens, it was observed that the accessory piece has a ridge, which separates the distal hook from the C-shaped part. This ridge continues towards the basal bulb as a filament. Most proximally, it forms a separate structure that attaches separately to the basal bulb. This ridge was also observed on one paratype of *C. reversati* on the slide RMCA37.402. However, the paratypes lost transparency due to the fixation in Malmberg’s and were difficult to observe in detail. On the seven other paratypes and vouchers, all collected by Pariselle & Euzet (2003) and deposited at the Royal Museum for Central Africa, Tervuren, Belgium, these structures were not observable. Because of this, the holotype, prepared with the same fixative, was not ordered from the Muséum national d’Histoire naturelle in Paris.

**Figure 10.**
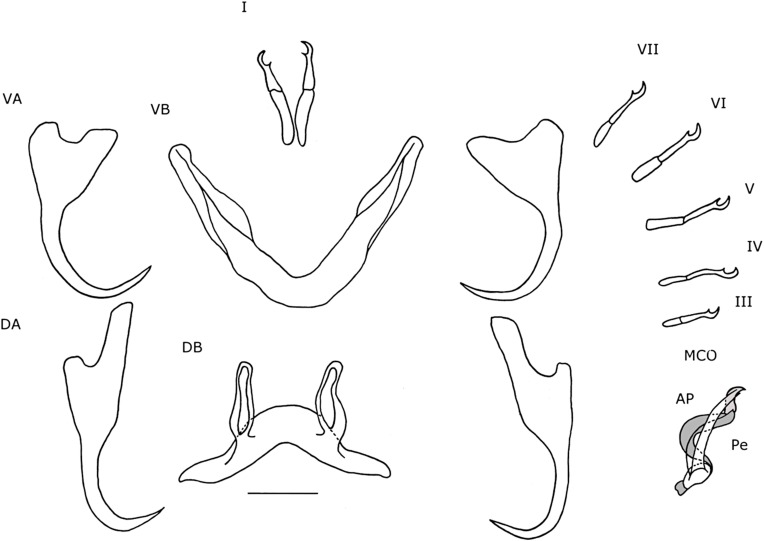
Haptoral and genital hard parts of *Cichlidogyrus reversati* from *Coptodon tholloni*. Accessory piece of the MCO in grey, to highlight the plate-like structure of the accessory piece. I–VII, hooks; AP, accessory piece; DA, dorsal anchors; DB, dorsal transverse bar; MCO, Male Copulatory Organ; Pe, penis; VA, ventral anchors; VB, ventral transverse bar. Scale bar: 20 μm.

### *Onchobdella ximenae* Jorissen, Pariselle, Kusters & Vanhove n. sp.

urn:lsid:zoobank.org:act:826D2ABF-DB22-459B-8006-3BC12CEB8A6A

Type host: *Hemichromis elongatus* (Guichenot, 1861).

Other hosts: *H. stellifer* Loiselle, 1979.

Infection site: Gills.

Type locality: Pond Near Kila Kindinga 5°29′7″S 14°53′3.8″E.

Other localities: Mvuazi River on *H. elongatus* 5°19′S; 15°7′E; Mbola River near Tshianya village on *H. stellifer* 05°52′09.8″S 12°39′52.6″E.

Material: Three whole-mounted specimens in Hoyer’s medium, including the holotype and 13 in Malmberg’s solution.

Holotype: M.T. 38311.

Paratypes: M.T. 38319–20, 38324–25, 38327–28, 38334NHMUK 2018.1.31.2 and 2018.1.31.4, KN10049 https://laji.fi/en/view?uri=luomus:KN.10049 and 10052 https://laji.fi/en/view?uri=luomus:KN.10052, SAMC-372 A090065, A090067.

Symbiotype: RMCA_Vert_2015.030.P.0020.

Paratype host vouchers: RMCA_Vert_2015.030.P.0019, RMCA_Vert_2015.030.P.0021, RMCA_Vert_2015.030.P.0022, AB53952197.

Etymology: The species epithet is a homage to Ximena Sariñana Rivera, a Mexican singer and is a noun (name) in the genitive case.

Authorship: Note that the authors of the new taxon are different from the authors of this paper; Article 50.1 and Recommendation 50A of International Code of Zoological Nomenclature [13].

#### Description (Table 5, Figs. 8e–8f, 11)

Dorsal anchors large (a = 51 μm) and orientated distal-laterally. Shaft undeveloped and bulbous (c = 2 μm). Guard with rounded edges. Point long with sudden and well-marked thickening on the interior side of the curve. Ventral anchors (f = 11 μm), with T-shaped base with rounded edges and a short, curved point. Dorsal bar slightly bent where both arms meet. Both arms make a 90° turn distally and thicken slightly. Distal tip of each arm bulbously thickened. Two ventral bars, thin, near straight. Six pairs of small hooks of same size. MCO consists of a basal bulb with a long, tubular penis (Pe = 66 μm), which makes one large loop of almost 360° along its course. A rounded, irregularly-shaped heel engulfs the basal bulb, except where the penis transitions into the basal bulb. Accessory piece consists of two rib-like structures that form an ellipsoid (resembling a windsurf wishbone), through which the penis passes. Both rib-like structures come together proximally, where they form a small bulge and distally, where their connection is smooth. At the distal end of the penis a leaf-like, smooth-edged plate is present, and is orientated perpendicular to the ellipsoid. Vagina, small, with two 90° curves, tubular (L = 33 μm).

**Figure 11.**
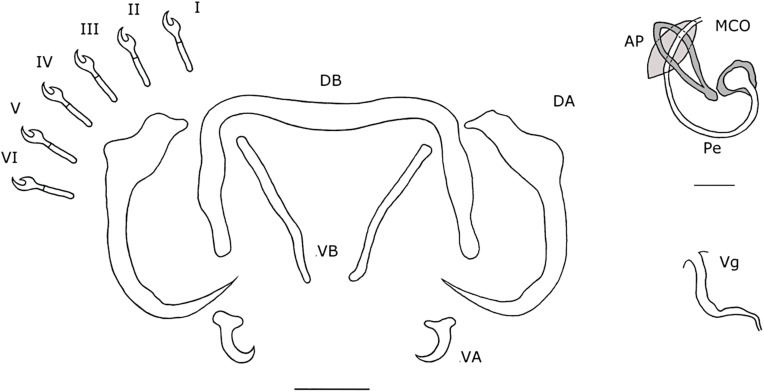
Haptoral and genital hard parts of *Onchobdella ximenae* n. sp. from *Hemichromis elongatus.* Accessory piece of the MCO in grey, to highlight the plate-like structure of the accessory piece. I-VI, hooks; AP, accessory piece; DA, dorsal anchors; DB, dorsal transverse bar; MCO, male copulatory organ; Pe, penis; VA, ventral anchors; VB, ventral transverse bars; Vg, vagina. Scale bar: 20 μm, scale bar of the MCO 10 μm.

**Table 5.** Measurements of *Onchobdella ximenae* n. sp. and *O. voltensis*. All measurements in μm as the average ± standard deviation, count and range (in parentheses).

Species	*O. ximenae* n. sp.	*O. voltensis*
Host	*H. stellifer, H. elongatus*	*H. fasciatus, H. bimaculatus*
Locality	4, 6 & 7	Volta Lake
Reference	Present study	Paperna [26]
Number of specimens	12	7
Ventral Anchor		
f	11, 5 (8–12)	15–20
Dorsal Anchor		
a	51, 6 (49–54)	60–90
b	43, 6 (40–46)	/
c	2, 6 (1–3)	/
d	19, 6 (17–21)	10–15
e	22, 6 (21–23)	/
Uncinuli		
uncinuli 1	/	10
uncinuli 2	13, 2 (12–13)	/
uncinuli 3	13, 3 (12–15)	/
uncinuli 4	13, 3 (12–14)	/
uncinuli 5	13, 4 (11–13)	/
uncinuli 6	13, 6 (11–15)	/
Ventral Bars		
x	34, 6 (29–43)	20–40
Dorsal Bar		
x	50, 5 (31–69)	70–100
h	33, 5 (29–41)	/
w	5, 6 (4–6)	2–5
MCO		
pe	66, 7 (60–70)	30–50
ap	22, 6 (19–26)	25–30
he	3, 7 (2–5)	N.A.
Vg		
L	33, 2 (21–40)	N.A.
l	3, 2 (2–3)	N.A.

#### Remarks

*Onchobdella ximenae* n. sp. resembles *O. voltensis* Paperna, 1968, described from Lake Volta, Ghana from *H. fasciatus* and *H. bimaculatus*. Both *O. ximenae* n. sp. and *O. voltensis* have ventral bars with solid distal ends instead of filamentous ends, such as are seen in all other congeners. Furthermore, both species have an accessory piece that forms an ellipsoid, a penis that passes through it and a smooth, leaf-like plate at the distal end of the accessory piece. The differences between these species are that the dorsal anchors in *O. ximenae* n. sp. are 10–40 μm smaller (see Table 5) and have an undeveloped shaft. The ventral anchors have a T-shaped base, contrary to the ones in *O. voltensis*, which have a base that is orientated proximally and not laterally. The penis of *O. voltensis* is J-shaped, whilst in *O. ximenae* n. sp. it almost makes a loop and is 16–36 μm longer (see Table 5). Also, the leaf-shaped plate is orientated perpendicularly on the ellipsoid, while in *O. voltensis* it follows the orientation of the ellipsoid. Lastly, *O. voltensis* does not have a sclerotized vagina.

## Discussion

Seventeen species of monogeneans, eight of which are new for science, were collected from five host species. Six of these new parasite species are described. For *C. reversati*, slight variations in morphology are mentioned as well as a ridge on the accessory piece. Two undescribed parasites, *C.* sp.1 and *C.* sp.2, were also found in the Middle Congo Basin and will be described in the report from that expedition.

### Parasites of *Coptodon*

From the Tondé estuary (see Fig. 1), the non-native *Coptodon rendalli* was captured [10]. Its native range includes tributaries and parts of the Middle and Upper Congo, the Zambezi, Cuanza, Catumbela, Okavango and Cunene rivers [10]. In Lower Congo, *Coptodon rendalli* was infected by *Cichlidogyrus berradae* Pariselle & Euzet, 2003; *Cichlidogyrus cubitus* Dossou, 1982 and *Cichlidogyrus flexicolpos* Pariselle & Euzet, 1995 (see Table 1). These species have not been found on *Coptodon rendalli* before, but on other representatives of *Coptodon* from Benin, Ivory Coast, Cabinda and the ROC [35]. Additionally, parasite species known from *Coptodon rendalli* from other regions, such as *Cichlidogyrus dossoui* Douëllou, 1993; *Cichlidogyrus quaestio* Douëllou, 1993; *Cichlidogyrus papernastrema* Price, Peebles & Bamford, 1969 or *Cichlidogyrus tiberianus* Paperna, 1960 [7, 14, 46] were not found in Lower Congo. This indicates that *Coptodon rendalli* lost its original parasite fauna during or after introduction, supporting the enemy-release hypothesis [38]. When comparing the parasite fauna of the native *Coptodon tholloni* with that of *Coptodon rendalli* in this study, only *C. cubitus* is shared between the two hosts. However, our sample size is small and, therefore, it is presumable that more parasite species are shared. *Cichlidogyrus cubitus* does not occur in the native range of *Coptodon rendalli*, so it is possible that *Coptodon rendalli* acquired this parasite through a spillback effect from *Coptodon tholloni* [11]. The same applies for *C. flexicolpos* on *Coptodon rendalli*. However, the reservoir for *C. flexicolpos* in Lower Congo could also be *Coptodon guineensis*, since there are no records of *C. flexicolpos* on *Coptodon tholloni*. Furthermore, *Coptodon guineensis* is known to host *C. flexicolpos* [29] and to occur in the mangroves of Lower Congo [10]. In our study, *Gyrodactylus chitandiri* Zahradníčková, Barson, Luus-Powell and Přikrylová, 2016 infected *Coptodon tholloni*, which constitutes a new host and geographical record and results in a disjunct distribution for *G. chitandiri*. The parasite is known from *Coptodon rendalli* and *Pseudocrenilabrus philander* (Weber, 1897) from Chirundu, Zambezi River and Lake Kariba [49], whilst it was lacking in Upper Congo on both hosts [14]. Instead, *Coptodon rendalli* was infected by *Gyrodactylus nyanzae* Paperna, 1973 in Upper Congo [14]. The disjunct distribution of *G. chitandiri* could be biased due to limited sampling in the past. From our observations, species of *Gyrodactylus* are far less prevalent (occurring on one out of 14 infected cichlid specimens in Lower Congo and on 1 out of 12 in Upper Congo) than species of *Cichlidogyrus* on gills of African cichlids, so it is easy to miss species of *Gyrodactylus* during sampling. We hypothesize that *G. chitandiri* shows a continuous distribution from the Zambezi Basin to Lower Congo. It should be noted that although their minimum prevalence is lower than that of species of *Cichlidogyrus*, their infection intensity is often higher (see Table 2 in this study and [14]). One specimen of *Coptodon tholloni* from the Ndimba Leta ponds, Mbanza-Ngungu (locality 3, Fig. 1) was exclusively infected by *Cichlidogyrus tilapiae* Paperna, 1960. This parasite is a generalist (see [21]), but has mostly been found on mouth-brooding cichlids (Oreochromini, see [8]) and has a wide native range that spans most of Central and western Africa, and the Levant [14, 27, 35]. Several species of *Coptodon* have been screened previously for parasites (e.g. *Coptodon rendalli* from Upper Congo [14, 46]), but of these, *C. tilapiae* has only been found on *Coptodon zillii*, so far [9]. In the case of the *C. tilapiae* infection on *Coptodon zillii*, *O. niloticus* was also present in the area [9], which is also the case in the Ndimba-Leta ponds. Whether the infection of *C. tilapiae* on *Coptodon tholloni* is natural or a result of spillover (see [45] and references therein) has to be determined in future research. If the infection is the result of spillover, this would support the hypothesis of Jorissen et al. [14], that certain aquatic systems, such as ponds or artificial lakes, stimulate interspecies interactions between cichlid hosts and have a higher host density, both of which stimulate parasite host-switching.

### Parasites of *Hemichromis*

The collected representatives of *Hemichromis* are the native *H. elongatus* and *H. stellifer* [10]. On these hosts, we have discovered three new species that are morphologically similar to already described ones: *O. ximenae* n. sp., which resembles *O. voltensis*; *C. calycinus* n. sp., which resembles *C. teugelsi* and *C. polyenso* n. sp., which resembles *C. euzeti* [35]. Furthermore, we can assume from the literature that *C. euzeti* is sympatric (occurs on the same individual host) with *C. longicirrus* in Benin, Cameroon and ROC, though not explicitly stated [6, 22, 34], while in Lower Congo, *C. longicirrus* is sympatric with *C. polyenso* n. sp. Similarly, *O. voltensis* and *O. aframae* are presumably sympatric in Benin, Cameroon, Senegal, Gambia, Mali and Ivory Coast [6, 26, 34], while in Lower Congo *O. aframae* is sympatric with *O. ximenae* n. sp. As shown, there are similarities between the parasite faunas of *Hemichromis* spp. throughout different ecoregions. However, parasites of *Hemichromis* spp. remain unexplored for large portions of Africa; thus, it is too early to draw conclusions about their biogeography and diversity. Nonetheless, it can be hypothesized that compared with the species discovered in Lower Congo, more morphologically similar species exist in other freshwater ecoregions on other representatives of *Hemichromis*. *Cichlidogyrus kmentovae* n. sp. was only found on *H. stellifer*, but our sample size is too small to verify whether it does not infect *H. elongatus* as well. *Cichlidogyrus falcifer* occurs on *H. fasciatus* [35] as well as on *H. elongatus* and thus is an intermediate specialist (a parasite occurring on more than one host from the same genus, see [21]).

### Parasites of *Tylochromis praecox*

From *Tylochromis praecox*, *C. bixlerzavalai* n. sp. and *C. omari* n. sp. are described. New species were expected as no monogeneans had yet been described from *T. praecox*. Furthermore, all dactylogyrids from species of *Tylochromis* mentioned in the literature are considered strict specialists [14, 23, 35, 36], meaning, they occur on a single host species. In those studies, representatives of *Tylochromis* were caught in low numbers and restricted to a single host species. In Lower Congo, *Tylochromis labrodon* Regan, 1920 and *Tylochromis lateralis* (Boulenger, 1898) occur further upstream and are sympatric, but not with *T. praecox* [43]. It would be worthwhile to investigate whether *C. bixlerzavalai* n. sp. and *C. omari* n. sp. occur on these hosts as well, to see if these parasites are all strict specialists or if they only appear to be so because of biogeographical barriers. The ancestral state for host-specificity within *Cichlidogyrus*/*Scutogyrus* is intermediate specialism [21]. Species of *Cichlidogyrus* that infect species of *Tylochromi*s are shown to be ancestral to all others within *Cichlidogyrus*/*Scutogyrus* [19–21, 37, 48], similar to *Tylochromis* (and Tylochromini) being ancestral to all African cichlids except *Heterochromis* [8, 43]. We could hence hypothesize that species of *Cichlidogyrus* that infect species of *Tylochromis* are intermediate specialists when sympatric host congeners are present in the area. It has to be noted that only *Cichlidogyrus pouyaudi* Pariselle & Euzet, 1994 was included as a representative that infects species of *Tylochromis* in the phylogenies of *Cichlidogyrus*/*Scutogyrus* [19–21, 37, 48]. However, we expect species of *Cichlidogyrus* that infect species of *Tylochromis* to form a monophyletic clade based on the morphological similarities between the species [37]. Both new species showed typical morphological characters of their congeners that infect species of *Tylochromis*, such as less developed auricles on the dorsal transverse bar, an accessory piece that does not connect directly to the basal bulb of the penis, and a spirally-winding penis [23, 28]. Following the phylogeny, we assume these morphological characters to be ancestral to all others within *Cichlidogyrus*/*Scutogyrus* [37].

### Species richness and comparison with other regions

Half of the parasite species found in this study are new to science, which is high in comparison to the single new species described from the Mweru-Luapula subregion in the Upper Congo Basin [14]. This can partially be explained by the Lower Congo region being more diverse and having many biogeographical barriers that facilitate speciation [15, 16]. In addition, almost all host species in the Lower Congo region were screened for parasites for the first time. Also, most of the cichlid species studied in the Mweru-Luapula subregion had a more widespread distribution than the ones in Lower Congo [10], and thus it was more likely for us to find previously-described parasites [14]. The diversity of monogenean parasites outmatches that of their hosts by more than 3:1 in this study, which is in line with the African average of 3.1 [44]. The parasite fauna reported in this study has more species in common with ecoregions from other basins, such as the nearby Ogooué-Nyanga-Kouilou-Niari ecoregion than with the rest of the Congo Basin. A possible explanation for this is that the Lower Congo was not a part of the Congo Basin until the Early Quaternary [42] and has stayed isolated from the rest of the Congo Basin because of the biogeographical barriers that the region is known for [15, 16, 24, 42]. Furthermore, representatives of *Tylochromis* and *Hemichromis* have predominantly been screened from Ghana, Benin, Cameroon, Senegal, Gambia, Mali, Guinea and Ivory Coast [6, 25, 26, 28, 34], whilst most parts of the Congo Basin or Central Africa remain unexplored. These genera have their origins in West Africa [18]. Furthermore, the parasite fauna of *P. cabrae* from the coastal lowlands of the ROC was reported to be highly homogeneous with that of West African cichlid hosts [33], so it is possible that an overlap in monogenean biodiversity between these two regions exists. Our results support this claim.

## Note

Michiel Jorissen won the prize for the best student presentation at the 8th International Symposium on Monogenea, held on 6–11 August 2017 at Masaryk University, Faculty of Science, Brno, Czech Republic, which was a fee-waiver for a paper in *Parasite*. The cost of publication of the present paper was thus offered by EDP Sciences.

## Conflict of interest

The authors declare that there is no conflict of interest.
